# Higher Serum Testosterone Level Was Associated with a Lower Risk of Prediabetes in US Adults: Findings from Nationally Representative Data

**DOI:** 10.3390/nu15010009

**Published:** 2022-12-20

**Authors:** Jason Wang, Alice F. Yan, Lawrence J. Cheskin, Zumin Shi

**Affiliations:** 1 Marriott’s Ridge High School, Marriottsville, MD 21104, USA; 2 Office of Research Patient Care Services, Stanford Health Care, Palo Alto, CA 94305, USA; 3 Department of Nutrition and Food Studies, George Mason University, Fairfax, VA 22030, USA; 4 Human Nutrition Department, College of Health Sciences, QU Health, Qatar University, Doha 2713, Qatar

**Keywords:** diabetes, prediabetes, testosterone

## Abstract

Low testosterone may be a novel risk factor for prediabetes. We assessed the associations between prediabetes and total serum testosterone (TT), and whether the associations were modified by population characteristics. The data from 5330 adults aged ≥ 20 years, who participated in the 2011–2016 National Health and Nutrition Examination Survey in the United States, were used. Prediabetes was based on fasting plasma glucose, HbA1c, or OGTT. Sociodemographic, obesity, co-morbidities, and lifestyle factors were included in logistic regression models. A dose-response relationship was found between prediabetes and the testosterone quartiles. The odds ratio (OR and 95% CI) for prediabetes across the quartiles of TT were: 1.00, 0.68 (0.50–0.92), 0.51 (0.36–0.72), and 0.48 (0.34–0.70) in men; and 1.00, 1.06 (0.81–1.40), 0.81 (0.61–1.06), and 0.68 (0.49–0.93) in women. The results changed marginally if the models were adjusted for additional variables such as BMI. The subgroup analyses showed differences in the association, which was stronger in some groups (for men: age < 50, white and black, overweight/obese, adequate physical activity, never-smoking; and for women: age ≥ 50, black). A higher testosterone level was associated with a lower risk of prediabetes among US adults. The strength of the association varied by population characteristics, weight status, gender, and lifestyle factors.

## 1. Introduction

Diabetes has become a serious global public health problem. Approximately 96 million US adults, or 1 in 3, have prediabetes [1]. Prediabetes is a condition characterized by elevated fasting blood sugar levels, hemoglobin A1c, or impaired glucose tolerance, but not high enough to be classified as diabetes [2,3]. People with prediabetes have an increased risk of developing type 2 diabetes (T2DM), heart disease, and stroke [4]. Without timely intervention, newly identified prediabetes is likely to progress to diabetes within three years [5], and up to 70% of people with prediabetes will develop diabetes within their lifetime [3].

Identifying risk factors for prediabetes is crucial for the early prevention of diabetes. Beyond traditional risk factors such as obesity, researchers have started to identify novel modifiable risk biomarkers, such as serum testosterone. Prior research shows that high testosterone levels are associated with a higher risk of T2DM in women, but with a lower risk in men [6,7,8,9]. These findings highlight the importance of deciphering the role of gender in the development of T2DM.

Although the evidence shows a higher percentage of men (41.0%) than women (32.0%) have prediabetes [1], serum testosterone as a novel risk biomarker for prediabetes has not been fully investigated in both genders. While some studies reported that serum testosterone predicted the risk of T2DM in men [10,11], fewer studies have investigated the relationship in adult women. In addition, consistently strong evidence shows racial/ethnic and socioeconomic status (SES) disparities in healthy lifestyle behaviors and diabetes outcomes. Therefore, it is critical to examine the association between prediabetes and serum testosterone, and how sex-specific associations are modified by population characteristics and lifestyle factors.

Previously published studies were limited by the study design and/or study sample. The majority are clinically-based studies or examine relatively small cohorts of specific ages and in limited regions [7,8]. The National Health and Nutrition Examination Survey (NHANES), a population-based survey, provides nationally-representative data that include comprehensive metrics on demographics, laboratory results, and examinations. Two previous studies using the NHANESIII (1988–1994) assessed the link between total testosterone levels and prediabetes [11,12]. In population studies, dietary patterns have been shown to be associated with testosterone levels [13,14]. The link between dietary patterns and diabetes/prediabetes has been well established [15,16,17]. As lifestyles and the prevalence of chronic diseases have changed substantially over the past three decades in the US, an updated analysis on the topic is needed. It has been reported that there has been a decline in testosterone levels over the past two decades in men in the USA [18]. Biomarkers are indicators of the biological mechanisms underlying health and disease. Efforts to optimize individual approaches to T2DM prevention that use novel biomarkers will help to shed light on how population subgroups, determined by population characteristics and lifestyle behaviors, may have differential risks for prediabetes.

Using nationally representative data collected in the United States, this study aimed to: (1) examine the association between testosterone levels and prediabetes in adults, and the differences in the association among population groups; and (2) test the interaction between testosterone levels and lifestyle factors in relation to prediabetes.

## 2. Materials and Methods

### 2.1. Study Design and Sample

Continuous NHANES is a cross-sectional survey started in 1999. It uses a multistage probability sampling technique to select a representative sample (~5000 each year) of the non-institutionalized population in the USA. It uses interviews, questionnaires, laboratory testing, and physical examination to collect the data. A detailed description of the methods used can be found elsewhere [19]. The interviews were conducted in the participants’ homes, while the physical examinations and blood sample collection were conducted at mobile examination centers. The National Centre for Health Statistics Institutional Ethics Review Board approved the study, and written consent was taken from all the participants. Continuous NHANES data are released in two-year cycles and are publicly available.

**Figure 1:**
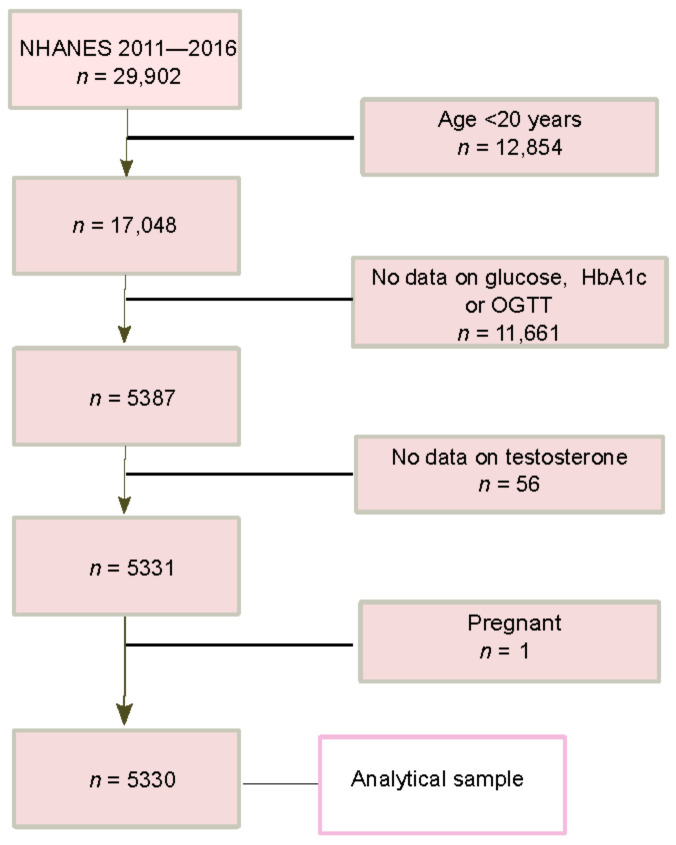
Flow chart of study sample selection of the final analysis.

The current analysis used data from three survey cycles (2011–2012, 2013–2014, and 2015–2016). From the 17,048 adults aged ≥ 20 years, 5330 adults were included in the analysis after excluding those without data on glucose, HbA1c or OGTT, testosterone, or being pregnant (Figure 1).

### 2.2. Key Study Outcome Variables: Diabetes and Prediabetes

Diabetes was defined as having fasting glucose ≥ 126 mg/dl, HbA1c ≥ 6.5%, or 2-hour plasma glucose ≥ 200 mg/dL during OGTT, or self-reported doctor-diagnosed diabetes [20]. Impaired fasting glucose was defined as fasting plasma glucose of 100 mg/dL to less than 126 mg/dL, IGT as 2-h plasma glucose of 140 mg/dL to less than 200 mg/dL, and an increased HbA_1c_ level as HbA1c level between 5.7% and 6.4%. Prediabetes was defined as having IFG, IGT, or increased HbA1c level (but less than 6.5%).

### 2.3. Key Study Exposure Variable: Testosterone

Overnight fasting blood samples were used to measure serum testosterone using the isotope-dilution liquid chromatography-tandem mass spectrometry (ID-LC-MS/MS) method for routine analysis developed by the CDC. It was created for high sample throughput and demonstrates high accuracy and precision over multiple years. The method was certified by the CDC Hormone Standardization Program (HoSt). The lower detection limit of the assay was 0.75 ng/mL. The details of the laboratory methodology for testosterone determination are available at: https://wwwn.cdc.gov/Nchs/Nhanes/2013-2014/TST_H.htm (accessed on 1 August 2022).

### 2.4. Covariates

The following variables were treated as covariates: age, sex, race (whites, blacks, Mexican Americans, other race), education level (lower than high school, graduated from high school or equivalent institution, any college, and college graduate or above), leisure time physical activity, smoking status (non-smoker, ex-smoker, and current smoker), alcohol intake, hypertension, and BMI. Age, sex, ethnicity, education level, smoking status, and alcohol intake were self-reported. The Poverty Income Ratio (PIR) was calculated by dividing the income of the family by the poverty threshold of the family and was categorized as <1.3 (low), 1.3–3.5 (moderate), and >3.5 (high) [21]. Hypertension was defined as systolic blood pressure ≥ 140 mmHg, diastolic blood pressure ≥ 90 mmHg, or a self-reported doctor’s diagnosis of hypertension, currently taking antihypertensive medications [22]. In the NHANES, food intake was assessed using a 24 h food recall for two days. On the first day, the food recall was conducted during a face-to-face interview in the Mobile Examination Center, and the second interview was conducted by a phone call 3–10 days later. We used the Healthy Eating Index (HEI) 2015 to reflect the quality of the overall diet based on the first-day food recall. The HEI-2015 was based on the 2015–2022 Dietary Guidelines for Americans, and nine adequacy and four moderation components [23]. The maximum score of the HEI-2015 was 100 points, with a high score representing better quality.

### 2.5. Statistical Analysis

Due to the significant sex difference in serum testosterone levels, the testosterone levels were categorized into sex-specific quartiles, with quartile 1 (Q1) and quartile 4 (Q4) as the lowest and highest. Sex-specific analyses were conducted. The sample characteristics were presented as the mean (SD) or as percentages; the differences in the subject characteristics were tested using a one-way analysis of variance (ANOVA) or chi-squared tests.

Multivariable logistic regression models were fit to examine the association between the quartiles of serum testosterone and prediabetes, controlling for different covariates and testing the differences across the groups. Six models were used: (1) model 1 was adjusted for age and race; (2) model 2 was further adjusted for race, the income-poverty ratio, physical activity, smoking, alcohol drinking, and family history of diabetes; (3) model 3 was model 2 further adjusted for the HEI-2015; (4) model 4 was model 2 further adjusted for hypertension; (5) model 5 was model 2 further adjusted for BMI as a continuous variable; and (6) model 6 was model 2 among those who did not use prescribed medications known to affect the hypothalamic–pituitary–gonadal (HPG) axis (including testosterone, antiandrogens, glucocorticoids, opiates, antiepileptics, or antipsychotics). These medications were extracted from the prescription medication dataset in each wave (dataset name RXQ_DRUG.xpt).

The variables included in the multivariable models were either socioeconomic factors or known risk factors for diabetes; and finally, subgroup analyses (by age, race, income to poverty ratio, education, smoking, hypertension, and overweight/obesity) were conducted to assess the association between serum testosterone and prediabetes.

The multiplicative interaction was tested by adding the product term of the two testing variables in the multivariable models. Survey weights, sample strata, and sample clusters were used in the multivariable analyses to account for the complex survey design. All the analyses were performed using STATA (Version 17.0, Stata Corporation, College Station, TX, USA).

## 3. Results

### 3.1. Study Sample Characteristics and Distribution of Health Outcomes

**Figure 2:**
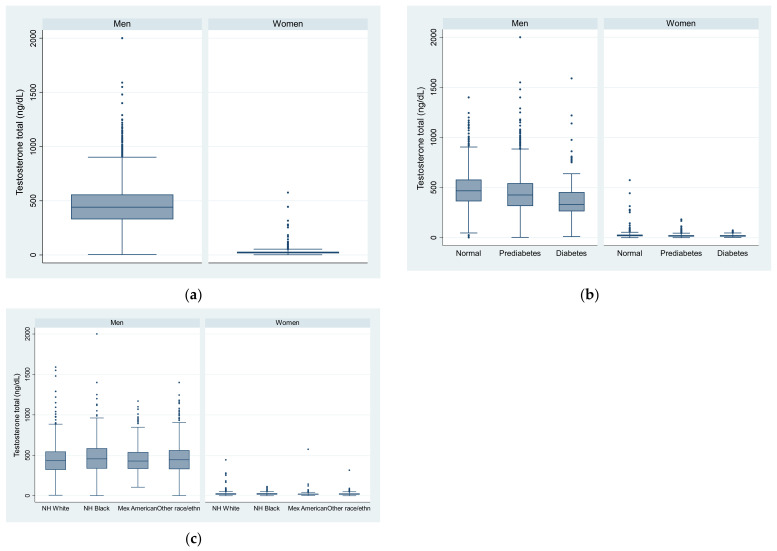
Box plot of testosterone level in US adults by sex, race/ethnicity, and diabetes status. (**a**) by sex; (**b**) by sex and diabetes status; (**c**) by sex and race/ethnicity.

The unweighted genomic mean (SD) testosterone was 418(SD = 1.6) (ng/dL) in men and 20.3 (1.8) in women (Table 1). Across the quartiles of testosterone, the mean age and prevalence of hypertension decreased, but the prevalence of smoking increased in both men and women. The BMI decreased with the increase of testosterone in men, but not in women. There was no significant difference in the HEI-2015 across the quartiles of testosterone in both men and women. The unweighted prevalence of prediabetes was 51.9% in men and 42.3% in women. Figure 2 shows the distribution of testosterone by sex, race/ethnicity, and diabetes status.

### 3.2. Association between Testosterone and Prediabetes

The unweighted prevalence of prediabetes across the quartiles of serum testosterone was 57.3%, 54.0%, 48.1%, and 48.0% in men; and 53.3%, 45.0%, 37.7%, and 33.4% in women, respectively. Serum testosterone was inversely associated with prediabetes in men and women (Table 2). After adjusting for age, race, income-poverty ratio, physical activity, smoking, alcohol drinking, and family history of diabetes, the odds ratio (OR, 95%CI) for prediabetes diabetes across the quartiles of serum testosterone were: 1.00, 0.73 (0.53–1.00), 0.57 (0.40–0.81), and 0.51 (0.35–0.72) (*p* for trend <0.001) in men; and 1.00, 1.13 (0.83–1.53), 0.82 (0.63–1.07), and 0.72 (0.52–1.01) (*p* for trend 0.013) in women. The association was not materially changed after further adjusting for BMI or hypertension in women. However, the association was attenuated and became statistically not significant by the adjustment for BMI in men. Adjusting for the HEI-2015 did not change the association in both men and women. When excluding those on medications known to affect the HPG axis, the above associations remained.

The association between serum testosterone and prediabetes was significant in men < 50 years, but not in those ≥ 50 years (p for interaction 0.097) (Table 3). The opposite was seen in women: the association was significant only among those above 50 years of age. There was no significant interaction between testosterone and race, income, education, smoking, hypertension, overweight/obesity, and physical activity. However, the subgroup analyses showed differences in the associations, which were stronger in some groups (for men: white and black, overweight/obese, adequate physical activity, never-smoking; for women: black).

Values are odds ratios (95%CI). Models adjusted for age, race, education, income-poverty ratio, physical activity, smoking, alcohol drinking, and family history of diabetes. Stratification variables were not adjusted in corresponding models.

## 4. Discussion

Our study found that testosterone was inversely associated with prediabetes in both men and women from a nationally representative sample of the US adult population. The associations varied by population characteristics, obesity status, and lifestyles such as smoking and physical activity. Our findings suggest a higher serum testosterone level is associated with a reduced prevalence of prediabetes, independent of lifestyles including the HEI-15. The findings are consistent with some existing studies that have assessed the association between testosterone levels and diabetes [7,8,9]. Several systematic reviews and meta-analyses on the association between testosterone levels and diabetes have been published [7,8,9,24]. In a meta-analysis of cross-sectional studies, the women with diabetes had significantly higher levels of testosterone than those without diabetes (mean difference, 6.1 ng/dL) [8]. However, in men with diabetes, the level of testosterone was lower than those without the condition (mean difference, −76.6 ng/dL) [8]. In cohort studies, the level of testosterone was also lower among those developed diabetes compared to those who did not develop diabetes [8]. A 2018 review based on 13 cohort or nested case–control studies found that higher total testosterone might reduce the risk of type 2 diabetes, with a RR of 0.65 (95% CI 0.50–0.84) [7]. A five-year follow-up study in men participating in the Men Androgen Inflammation Lifestyle Environment and Stress (MAILES) Study reported that low testosterone predicted the incidence of type 2 diabetes, independent of current risk models [10]. A cut-off of <16 mnol/L for low serum testosterone had a positive predictive value of 12.9% for type 2 diabetes risk.

A few studies have examined the association between testosterone levels and prediabetes [11,12]. Overall, they suggest an inverse association between the two, although some results varied. For example, in NHANES III, the total testosterone concentration was lower among men with prediabetes (4.89 ng/mL) than men without prediabetes/diabetes (5.29 ng/mL) [12]. Men with prediabetes were more likely to have low testosterone (OR 2.58, 95%CI 1.54–4.29) [11]. In clinical trials, testosterone treatment has been shown to prevent type 2 diabetes in men. For example, in the T4DM (testosterone for the prevention of type 2 diabetes) trial, testosterone treatment for two years reduced the risk of type 2 diabetes beyond the effects of a lifestyle program. The trial recruited 1007 men aged 50–74 years with a waist circumference >94 cm, plus either IGT or newly diagnosed T2DM. In the testosterone-treated group, there was a 40% reduction in the prevalence of T2DM after two years compared with the placebo group. In our study, comparing the extreme quartiles of serum testosterone, the likelihood of having prediabetes was 49% and 28% lower in men and women, respectively.

Although testosterone levels decrease with aging, one of the main drivers of the decline is obesity, according to previous research [25,26,27]. It is interesting that the inverse association between testosterone levels and prediabetes was only significant among men with obesity. This may suggest that, for men with normal body weight, testosterone should not be used to prevent T2DM.

Fewer studies have examined the association between testosterone levels and diabetes in women than in men [8]. Unlike men, the findings from a systematic review suggest that high testosterone levels are positively associated with the risk of diabetes in women [8]. However, most of the studies included in the review examined fewer than 100 diabetes cases. In our study, testosterone levels were inversely associated with prediabetes, independent of lifestyle factors, obesity, and hypertension. However, the association varied by age and was only significant among those over 50 years old. Importantly, comparing the extreme quartiles of testosterone for women over 50, the OR for diabetes was 0.57 (95% CI 0.37–0.90), suggesting a 43% decreased likelihood of having diabetes. For those < 50 years, Q2 to Q4 of testosterone levels had ORs greater than 1, although not statistically significant. Further studies are needed to investigate this difference.

The mechanisms underlying the association between testosterone and glucose metabolism have been proposed but remain uncertain. First, there is an inverse association between testosterone and insulin resistance [28,29]. Second, testosterone regulates the expression of genes involved in insulin signaling and glucose uptake, e.g., glucose transporter-4 (GLUT4). Third, obesity is a major driver of age-related testosterone decline [25,26,27]. Another important consideration is that modern lifestyles may affect our circadian rhythm. There are several consequences of circadian disruption, including sleep disorders, metabolic syndrome, and obesity. These conditions have been shown to be related to both low testosterone and a high risk of diabetes [24]. It is worth mentioning that plasma testosterone levels vary in a circadian manner: the highest upon waking and decreasing to a low point at the end of the day [30]. Low testosterone may affect overall sleep quality, which could subsequently increase the risk of diabetes [31].

The age and sex differences in the associations between serum testosterone levels and prediabetes were unexpected. It could be due to the ratio of testosterone and estrogen in men and women and age-related changes in the ratio. A study conducted in China found that the ratio between testosterone and estradiol has been found to be inversely associated with cardiovascular diseases in men, but not in women [32]. Further research is needed to elucidate the mechanisms.

This study has some key strengths. First, it used three cycles of NHANES data with 5330 men and women. The study sample is representative of the general US adult population; therefore, the findings are generalizable. In addition, detailed sociodemographic and lifestyle factors were available and allowed us to not only adjust for confounders but also to conduct subgroup analyses. We were able to identify those who used prescription medications known to affect the HPG axis. In the sensitivity analyses, excluding those medication users did not change the findings. This study also has some limitations. The cross-sectional study design cannot test causation, as reverse causation is possible. Another limitation is the lack of detailed information on treatment for all the participants with testosterone or other medications that could affect testosterone levels, such as the dose and duration of the medications’ use.

## 5. Conclusions

High testosterone was inversely associated with prediabetes. The associations varied by population characteristics, weight status, and lifestyle factors. Sex-specific differences exist in these associations. For example, the association between serum testosterone and prediabetes was significant in men < 50 years, but not in those > 50 years; the opposite was seen in women: the association was significant only among those above 50 years of age. Future prospective studies are needed to test causality and elucidate the biological mechanisms of the observed association and sex differences.

## Figures and Tables

**Table 1:** Sample characteristics by quartiles (Q1–Q4 high level) of testosterone among US men and women—NHANES 2011–2016 (*n* = 5330).

	Total	Q1	Q2	Q3	Q4	*p* Value
A. Men	*n* = 2633	*n* = 661	*n* = 657	*n* = 657	*n* = 658	
Total testosterone, mean (SD) (ng/dL) *	418.3 (×/1.6)	230.8 (×/1.7)	383.9 (×/1.1)	493.0 (×/1.1)	703.2 (×/1.2)	<0.001
Total testosterone, mean (SD) (ng/dL)	461.4 (193.6)	249.8 (68.2)	385.3 (33.0)	494.1 (33.3)	717.5 (160.2)	<0.001
Age, mean (SD) (years)	47.5 (17.3)	50.9 (17.3)	47.6 (17.3)	46.5 (16.7)	45.1 (17.4)	<0.001
Education						0.091
<11 grade	591 (22.4%)	138 (20.9%)	160 (24.4%)	141 (21.5%)	152 (23.1%)	
High school diploma or GED	616 (23.4%)	150 (22.7%)	140 (21.3%)	148 (22.5%)	178 (27.1%)	
College	732 (27.8%)	202 (30.6%)	170 (25.9%)	184 (28.0%)	176 (26.7%)	
>Post-graduate School	694 (26.4%)	171 (25.9%)	187 (28.5%)	184 (28.0%)	152 (23.1%)	
Race						0.21
NH White	1096 (41.6%)	289 (43.7%)	280 (42.6%)	275 (41.9%)	252 (38.3%)	
NH Black	466 (17.7%)	109 (16.5%)	105 (16.0%)	117 (17.8%)	135 (20.5%)	
Mex American	356 (13.5%)	84 (12.7%)	103 (15.7%)	89 (13.5%)	80 (12.2%)	
Other race/ethnicity	715 (27.2%)	179 (27.1%)	169 (25.7%)	176 (26.8%)	191 (29.0%)	
Income to poverty ratio						0.23
<1.30	754 (31.1%)	173 (28.5%)	187 (30.8%)	182 (30.2%)	212 (34.8%)	
1.3–3.5	879 (36.2%)	218 (35.9%)	225 (37.0%)	217 (36.0%)	219 (35.9%)	
>3.5	794 (32.7%)	216 (35.6%)	196 (32.2%)	203 (33.7%)	179 (29.3%)	
Smoking						<0.001
Never	1240 (47.2%)	308 (46.7%)	321 (48.9%)	327 (49.9%)	284 (43.2%)	
Former	767 (29.2%)	229 (34.7%)	205 (31.2%)	169 (25.8%)	164 (25.0%)	
Current smoker	622 (23.7%)	123 (18.6%)	131 (19.9%)	159 (24.3%)	209 (31.8%)	
Drinking alcohol						0.011
No	385 (14.6%)	126 (19.1%)	95 (14.5%)	84 (12.8%)	80 (12.2%)	
Yes	1941 (73.7%)	457 (69.1%)	492 (74.9%)	494 (75.2%)	498 (75.7%)	
Missing	307 (11.7%)	78 (11.8%)	70 (10.7%)	79 (12.0%)	80 (12.2%)	
Physical activity (METs minutes/week)						<0.001
<600	778 (29.6%)	234 (35.5%)	182 (27.7%)	195 (29.7%)	167 (25.4%)	
600–1200	259 (9.8%)	75 (11.4%)	69 (10.5%)	65 (9.9%)	50 (7.6%)	
≥1200	1594 (60.6%)	351 (53.2%)	405 (61.7%)	397 (60.4%)	441 (67.0%)	
HEI-2015	41.9 (14.4)	41.8 (13.5)	42.3 (14.9)	41.7 (14.9)	41.6 (14.3)	0.86
BMI (kg/m2), mean (SD)	28.3 (5.9)	31.7 (6.7)	29.0 (5.1)	27.3 (4.8)	25.1 (4.4)	<0.001
Overweight/obese	1829 (69.9%)	569 (86.7%)	525 (80.3%)	442 (67.7%)	293 (44.9%)	<0.001
Hypertension	824 (31.8%)	270 (41.5%)	228 (35.2%)	186 (28.6%)	140 (21.7%)	<0.001
Diabetes status (IFG/A1C/OGTT)						<0.001
Normal	1024 (38.9%)	176 (26.6%)	240 (36.5%)	295 (44.9%)	313 (47.6%)	
Prediabetes	1366 (51.9%)	379 (57.3%)	355 (54.0%)	316 (48.1%)	316 (48.0%)	
Diabetes	243 (9.2%)	106 (16.0%)	62 (9.4%)	46 (7.0%)	29 (4.4%)	
Having known prediabetes	145 (5.7%)	50 (8.0%)	37 (5.8%)	29 (4.6%)	29 (4.5%)	0.022
Perceived at risk for diabetes/prediabetes	678 (26.0%)	200 (30.8%)	168 (25.8%)	165 (25.3%)	145 (22.3%)	0.005
Glucose test past 3 years	1171 (45.0%)	346 (53.3%)	286 (43.9%)	287 (44.0%)	252 (38.7%)	<0.001
Family history of diabetes	483 (18.3%)	139 (21.0%)	121 (18.4%)	125 (19.0%)	98 (14.9%)	0.035
Medicine use **	230 (8.7%)	68 (10.3%)	50 (7.6%)	53 (8.1%)	59 (9.0%)	0.33
**B. Women**	*n* = 2697	*n* = 676	*n* = 673	*n* = 674	*n* = 674	
Testosterone total (ng/dL) *	20.3 (×/1.8)	9.7 (×/1.5)	17.5 (×/1.1)	24.6 (×/1.1)	41.2 (×/1.4)	<0.001
Testosterone total (ng/dL)	24.4 (22.4)	10.3 (2.9)	17.6 (1.8)	24.7 (2.4)	45.3 (36.1)	<0.001
Age (years)	47.9 (17.2)	55.5 (15.4)	49.1 (15.8)	44.3 (16.2)	42.5 (18.1)	<0.001
Education						<0.001
<11 grade	543 (20.1%)	163 (24.1%)	141 (21.0%)	128 (19.0%)	111 (16.5%)	
High school diploma or GED	530 (19.7%)	130 (19.2%)	147 (21.8%)	125 (18.5%)	128 (19.0%)	
College	864 (32.0%)	190 (28.1%)	199 (29.6%)	216 (32.0%)	259 (38.5%)	
>Post-graduate School	759 (28.2%)	193 (28.6%)	186 (27.6%)	205 (30.4%)	175 (26.0%)	
Race						<0.001
NH White	1090 (40.4%)	259 (38.3%)	286 (42.5%)	257 (38.1%)	288 (42.7%)	
NH Black	530 (19.7%)	120 (17.8%)	109 (16.2%)	143 (21.2%)	158 (23.4%)	
Mex American	372 (13.8%)	99 (14.6%)	109 (16.2%)	96 (14.2%)	68 (10.1%)	
Other race/ethnicity	705 (26.1%)	198 (29.3%)	169 (25.1%)	178 (26.4%)	160 (23.7%)	
Income to poverty ratio						0.15
<1.30	868 (34.9%)	213 (35.0%)	198 (32.6%)	211 (33.2%)	246 (38.5%)	
1.3–3.5	888 (35.7%)	212 (34.9%)	218 (35.9%)	226 (35.6%)	232 (36.3%)	
>3.5	733 (29.4%)	183 (30.1%)	191 (31.5%)	198 (31.2%)	161 (25.2%)	
Smoking						<0.001
Never	1802 (66.9%)	477 (70.8%)	466 (69.3%)	446 (66.2%)	413 (61.3%)	
Former	453 (16.8%)	124 (18.4%)	107 (15.9%)	112 (16.6%)	110 (16.3%)	
Current smoker	439 (16.3%)	73 (10.8%)	99 (14.7%)	116 (17.2%)	151 (22.4%)	
Drinking						<0.001
No	384 (14.2%)	127 (18.8%)	92 (13.7%)	93 (13.8%)	72 (10.7%)	
Yes	1603 (59.4%)	356 (52.7%)	393 (58.4%)	411 (61.0%)	443 (65.7%)	
Missing	710 (26.3%)	193 (28.6%)	188 (27.9%)	170 (25.2%)	159 (23.6%)	
Physical activity (METs minutes/week)						0.085
<600	1159 (43.0%)	319 (47.2%)	288 (42.8%)	289 (42.9%)	263 (39.0%)	
600–1200	334 (12.4%)	86 (12.7%)	78 (11.6%)	85 (12.6%)	85 (12.6%)	
≥1200	1204 (44.6%)	271 (40.1%)	307 (45.6%)	300 (44.5%)	326 (48.4%)	
HEI-2015	43.5 (14.2)	44.4 (13.8)	43.5 (14.8)	43.5 (13.8)	42.7 (14.4)	0.22
BMI (kg/m^2^)	29.2 (7.6)	29.1 (7.2)	29.4 (7.1)	29.2 (7.8)	29.1 (8.1)	0.89
Overweight/obese	1803 (67.3%)	444 (66.4%)	467 (69.7%)	453 (67.3%)	439 (65.6%)	0.41
Hypertension	816 (31.1%)	287 (43.4%)	196 (30.2%)	159 (24.1%)	174 (26.6%)	<0.001
Type 2 Diabetes (IFG/A1C/OGTT)						<0.001
Normal	1320 (48.9%)	240 (35.5%)	301 (44.7%)	378 (56.1%)	401 (59.5%)	
Prediabetes	1142 (42.3%)	360 (53.3%)	303 (45.0%)	254 (37.7%)	225 (33.4%)	
Diabetes	235 (8.7%)	76 (11.2%)	69 (10.3%)	42 (6.2%)	48 (7.1%)	
Known prediabetes	217 (8.4%)	68 (10.6%)	51 (7.9%)	49 (7.6%)	49 (7.4%)	0.12
Perceived at risk for diabetes/prediabetes	870 (32.8%)	211 (31.7%)	219 (33.3%)	219 (33.0%)	221 (33.1%)	0.93
Glucose test past 3 years	1417 (53.4%)	409 (61.5%)	352 (53.5%)	349 (52.6%)	307 (46.0%)	<0.001
Family history of diabetes	674 (25.0%)	161 (23.8%)	172 (25.6%)	174 (25.8%)	167 (24.8%)	0.83
Medication use **	247 (9.2%)	94 (13.9%)	58 (8.6%)	48 (7.1%)	47 (7.0%)	<0.001

* geometric mean (SD) ** medication known to affect HPG axis (including testosterone, antiandrogens, glucocorticoids, opiates, antiepileptics, or antipsychotics).

**Table 2:** Odds ratio (95% CI) for prediabetes by quartiles (Q1–Q4 high level) of testosterone in US men and women—NHANES 2011–2016.

	Quartiles of Testosterone				*p*-Value
	Q1	Q2	Q3	Q4	
**Men**					
Model 1	1.00	0.68 (0.50–0.92)	0.51 (0.36–0.72)	0.48 (0.34–0.70)	<0.001
Model 2	1.00	0.73 (0.53–1.00)	0.57 (0.40–0.81)	0.51 (0.35–0.72)	<0.001
Model 2+HEI-2015	1.00	0.72 (0.52–1.00)	0.56 (0.39–0.80)	0.50 (0.35–0.73)	<0.001
Model 2+hypertension	1.00	0.75 (0.54–1.04)	0.59 (0.41–0.84)	0.52 (0.36–0.74)	<0.001
Model 2+ BMI	1.00	0.82 (0.59–1.15)	0.71 (0.48–1.05)	0.69 (0.45–1.05)	0.052
Model 2+excluding medication users **	1.00	0.68 (0.49–0.95)	0.53 (0.37–0.76)	0.46 (0.31–0.67)	<0.001
**Women**					
Model 1	1.00	1.06 (0.81–1.40)	0.81 (0.61–1.06)	0.68 (0.49–0.93)	0.004
Model 2	1.00	1.13 (0.83–1.53)	0.82 (0.63–1.07)	0.72 (0.52–1.01)	0.013
Model 2+HEI-2015	1.00	1.14 (0.84–1.55)	0.81 (0.62–1.05)	0.72 (0.51–1.02)	0.013
Model 2+hypertension	1.00	1.12 (0.84–1.50)	0.85 (0.65–1.10)	0.72 (0.52–0.99)	0.015
Model 2+ BMI	1.00	1.07 (0.78–1.49)	0.79 (0.60–1.05)	0.70 (0.50–0.98)	0.009
Model 2+excluding medication users **	1.00	1.12 (0.81–1.54)	0.84 (0.65–1.09)	0.72 (0.51–1.02)	0.014

** Those with medications known to affect HPG axis (including testosterone, antiandrogens, glucocorticoids, opiates, antiepileptics, or antipsychotics) were excluded.

**Table 3:** Subgroup analyses of the association between quartiles of testosterone and prediabetes in US men and women—NHANES 2011–2016.

	Quartiles of Testosterone				*p* Trend	*p* Interaction
	Q1	Q2	Q3	Q4		
**A. Men** Age ≥ 50 years						0.097
No	1.00	0.54 (0.36–0.81)	0.43 (0.29–0.64)	0.39 (0.26–0.59)	<0.001	
Yes	1.00	1.30 (0.69–2.43)	0.96 (0.50–1.86)	0.69 (0.39–1.23)	0.112	
Race						0.981
NH White	1.00	0.70 (0.46–1.07)	0.59 (0.36–0.96)	0.50 (0.31–0.80)	0.002	
NH Black	1.00	0.71 (0.38–1.33)	0.48 (0.26–0.91)	0.48 (0.26–0.89)	0.016	
Mex American	1.00	1.20 (0.46–3.14)	0.58 (0.24–1.40)	0.59 (0.28–1.25)	0.040	
Other race/ethnicity	1.00	0.68 (0.32–1.42)	0.58 (0.32–1.05)	0.53 (0.27–1.02)	0.052	
Income to poverty ratio						0.560
<1.30	1.00	0.45 (0.25–0.81)	0.39 (0.21–0.75)	0.34 (0.18–0.64)	0.003	
1.3–3.5	1.00	0.88 (0.49–1.58)	0.75 (0.46–1.21)	0.59 (0.35–1.00)	0.027	
>3.5	1.00	0.67 (0.36–1.26)	0.51 (0.29–0.89)	0.44 (0.26–0.75)	0.001	
Education						0.186
<11 grade	1.00	0.73 (0.29–1.82)	0.60 (0.30–1.23)	0.28 (0.13–0.60)	0.001	
HS diploma or GED	1.00	0.39 (0.18–0.83)	0.32 (0.13–0.79)	0.40 (0.18–0.88)	0.040	
College	1.00	0.67 (0.41–1.09)	0.57 (0.36–0.92)	0.68 (0.39–1.18)	0.120	
>Post-graduate school	1.00	1.13 (0.63–2.04)	0.74 (0.37–1.49)	0.51 (0.26–0.99)	0.027	
Smoking						0.204
Never	1.00	0.73 (0.45–1.17)	0.47 (0.30–0.73)	0.40 (0.24–0.65)	<0.001	
Former	1.00	0.88 (0.49–1.55)	0.74 (0.39–1.41)	0.50 (0.25–0.99)	0.039	
Current smoker	1.00	0.51 (0.24–1.08)	0.63 (0.28–1.44)	0.73 (0.34–1.55)	0.734	
Hypertension						0.707
No	1.00	0.78 (0.55–1.10)	0.61 (0.43–0.85)	0.56 (0.36–0.85)	0.002	
Yes	1.00	0.72 (0.34–1.50)	0.52 (0.24–1.10)	0.39 (0.20–0.76)	0.004	
Overweight/obese						0.866
No	1.00	0.67 (0.25–1.84)	0.61 (0.25–1.53)	0.61 (0.25–1.49)	0.354	
Yes	1.00	0.74 (0.54–1.03)	0.59 (0.41–0.84)	0.50 (0.32–0.79)	<0.001	
Physical activity (METs minutes/week)						0.804
<600	1.00	0.93 (0.41–2.09)	0.48 (0.21–1.09)	0.42 (0.20–0.89)	0.005	
600–1200	1.00	0.91 (0.28–3.01)	0.73 (0.24–2.21)	0.66 (0.17–2.61)	0.451	
≥1200	1.00	0.64 (0.37–1.09)	0.59 (0.38–0.91)	0.51 (0.31–0.84)	0.006	
**A. Women** Age ≥ 50 years						0.306
No	1.00	1.56 (0.91–2.69)	1.28 (0.77–2.12)	1.15 (0.64–2.06)	0.858	
Yes	1.00	1.01 (0.65–1.58)	0.60 (0.39–0.92)	0.57 (0.37–0.90)	0.002	
Race						0.464
NH White	1.00	1.43 (0.95–2.15)	0.98 (0.67–1.44)	0.96 (0.57–1.64)	0.438	
NH Black	1.00	0.88 (0.38–2.00)	0.62 (0.33–1.19)	0.45 (0.22–0.91)	0.010	
Mex American	1.00	0.65 (0.24–1.76)	0.53 (0.20–1.39)	0.68 (0.26–1.77)	0.316	
Other race/ethnicity	1.00	0.71 (0.42–1.17)	0.72 (0.41–1.25)	0.52 (0.29–0.93)	0.039	
Income to poverty ratio						0.254
<1.30	1.00	0.90 (0.49–1.65)	0.91 (0.63–1.30)	0.72 (0.44–1.20)	0.197	
1.3–3.5	1.00	1.18 (0.66–2.11)	1.02 (0.64–1.62)	0.87 (0.49–1.55)	0.480	
>3.5	1.00	1.50 (0.85–2.64)	0.69 (0.35–1.35)	0.80 (0.40–1.61)	0.133	
Education						0.285
<11 grade	1.00	0.76 (0.36–1.59)	0.57 (0.30–1.07)	0.63 (0.28–1.41)	0.140	
HS diploma or GED	1.00	1.21 (0.57–2.56)	1.50 (0.74–3.04)	0.77 (0.30–1.93)	0.627	
Some college	1.00	1.49 (0.83–2.69)	1.08 (0.55–2.10)	0.92 (0.54–1.59)	0.401	
>College	1.00	1.20 (0.62–2.33)	0.58 (0.35–0.96)	0.82 (0.42–1.61)	0.111	
Smoking						0.525
Never	1.00	0.90 (0.60–1.35)	0.81 (0.54–1.22)	0.72 (0.46–1.14)	0.112	
Former	1.00	2.64 (1.06–6.58)	0.97 (0.51–1.87)	0.83 (0.36–1.95)	0.181	
Current smoker	1.00	1.58 (0.56–4.49)	0.88 (0.38–2.04)	1.02 (0.38–2.72)	0.698	
Hypertension						0.679
No	1.00	1.11 (0.81–1.52)	0.86 (0.63–1.19)	0.81 (0.55–1.19)	0.117	
Yes	1.00	1.41 (0.70–2.84)	0.90 (0.46–1.74)	0.71 (0.35–1.44)	0.215	
Overweight/obese						0.376
No	1.00	0.79 (0.40–1.56)	0.69 (0.35–1.34)	0.79 (0.39–1.60)	0.428	
Yes	1.00	1.31 (0.84–2.06)	0.94 (0.63–1.40)	0.78 (0.51–1.20)	0.082	
Physical activity (METs minutes/week)						0.259
<600	1.00	1.09 (0.64–1.85)	0.88 (0.60–1.30)	0.99 (0.61–1.60)	0.704	
600–1200	1.00	1.13 (0.36–3.53)	0.91 (0.35–2.36)	0.53 (0.15–1.92)	0.267	
≥1200	1.00	1.23 (0.73–2.07)	0.76 (0.46–1.26)	0.69 (0.42–1.15)	0.061	

## Data Availability

The data used in the study are publicly available from the NHANES website.
